# Comment on “Intermittent Hypoxic–Hyperoxic Training During Inpatient Rehabilitation Improves Exercise Capacity and Functional Outcome in Patients With Long Covid: Results of a Controlled Clinical Pilot Trial” by Doehner et al.

**DOI:** 10.1002/jcsm.13802

**Published:** 2025-04-21

To the Editors of the Journal of Cachexia, Sarcopenia and Muscle

It is estimated that around 10% of people infected with COVID‐19 continue to struggle with permanent or new symptoms even months after the acute illness [1]. One of the most severe forms of this Long Covid syndrome is known as myalgic encephalitis/chronic fatigue syndrome (ME/CFS). This is a neuroimmunological disease with a prevalence in Germany of around 0.6% after COVID disease, which often leads to incapacity for work and disability [2]. I was therefore very interested to read a therapy study in which Long Covid patients were treated with ‘Intermittent Hypoxic–Hyperoxic Training’ (IHHT), three treatments per week for approximately 5 weeks [3], which resulted in a significant improvement in performance. The existence of such a method, which earlier publications suggest is even low in side effects, will raise hopes among the many thousands of chronically ill Long Covid patients or ME/CFS sufferers, as effective treatment options have not existed to date.

Unfortunately, doubts have arisen as to the quality of the study and also the accuracy of its conclusions. Allow us to briefly explain our doubts below, focussing on the primary endpoint of the study.

The authors formed two groups of 70 and 75 patients from the population of patients at a rehabilitation centre. Group allocation was not randomised. The 6‐min walking test was chosen as the primary endpoint as a measure of functional capacity. The baseline parameter was 352 ± 75 m in the study group and 430 ± 81 m in the control group. This corresponds to a difference of 25%. Statistically, this difference is highly significant with *p* < 0.001, that is, the probability that both groups come from the same collective is less than 1:100 000. Other performance parameters also differed significantly. It is therefore clear that different collectives are being compared here.

After the therapy, the IHHT group achieved a 6‐min walking test value of 443 ± 77 m, the control group 462 ± 89 m. You do not have to be a statistics expert to be able to say with great certainty that these two values do not differ significantly from each other. However, the training effect, that is, the difference between baseline value and the endpoint reached, is significantly greater in the IHHT group. The authors then conclude: ‘Respiratory treatment with IHHT in addition to a multidisciplinary rehabilitation programme improves functional capacity, symptomatic status and quality of life in patients with disabling Long Covid’.

In our opinion, this conclusion cannot be substantiated by the study. Due to the lack of a real randomised control group that was subjected to a sham treatment, for example, it cannot be ruled out that it was the standard rehabilitation measures alone that led to such a strong increase in performance in the study group that the two groups no longer differed at the end of the observation period. This is not surprising, as a training‐related increase in performance is of course also dependent on the initial training condition of the test person—and this is where the two groups differed significantly. To illustrate this with an example, an untrained person undergoing an intensive running training for several weeks is certainly able to achieve a time improvement of 1 s over 100 m. High‐performance athletes train for years and have problems improving the record over this distance by 0.1 s.

Accordingly, we believe that the design of the study does not allow any conclusions to be drawn. This is extremely regrettable. The tested procedure theoretically has the potential to improve the situation of Long Covid and ME/CFS patients. However, in our opinion, the elaborately conducted study was not designed to produce a reliable result and to substantiate the hope in the procedure used. Even if the authors point out at the end that their results require verification in controlled studies, the reference to ‘beneficial’ and ‘promising’ results appears premature and is associated with the risk of raising false hopes in ME/CFS patients who have not yet been offered any therapeutic options.
